# MITF controls the TCA cycle to modulate the melanoma hypoxia response

**DOI:** 10.1111/pcmr.12802

**Published:** 2019-07-08

**Authors:** Pakavarin Louphrasitthiphol, Ioanna Ledaki, Jagat Chauhan, Paola Falletta, Robert Siddaway, Francesca M. Buffa, David R. Mole, Tomoyoshi Soga, Colin R. Goding

**Affiliations:** ^1^ Ludwig Institute for Cancer Research, Nuffield Department of Clinical Medicine University of Oxford Oxford UK; ^2^ Department of Oncology University of Oxford Oxford UK; ^3^ Target Discovery Institute, Nuffield Department of Clinical Medicine University of Oxford Oxford UK; ^4^ Institute for Advanced Biosciences Keio University Yamagata Japan

**Keywords:** genomewide, glucose limitation, hypoxia, melanoma, MITF

## Abstract

In response to the dynamic intra‐tumor microenvironment, melanoma cells adopt distinct phenotypic states associated with differential expression of the microphthalmia‐associated transcription factor (MITF). The response to hypoxia is driven by hypoxia‐inducible transcription factors (HIFs) that reprogram metabolism and promote angiogenesis. HIF1α indirectly represses MITF that can activate HIF1α expression. Although HIF and MITF share a highly related DNA‐binding specificity, it is unclear whether they co‐regulate subset of target genes. Moreover, the genomewide impact of hypoxia on melanoma and whether melanoma cell lines representing different phenotypic states exhibit distinct hypoxic responses is unknown. Here we show that three different melanoma cell lines exhibit widely different hypoxia responses with only a core 23 genes regulated in common after 12 hr in hypoxia. Surprisingly, under hypoxia MITF is transiently up‐regulated by HIF1α and co‐regulates a subset of HIF targets including *VEGFA*. Significantly, we also show that MITF represses itself and also regulates SDHB to control the TCA cycle and suppress pseudo‐hypoxia. Our results reveal a previously unsuspected role for MITF in metabolism and the network of factors underpinning the hypoxic response in melanoma.

**Significance**

In contrast to expectations, our results suggest the early response to hypoxia includes a transient direct up‐regulation of MITF by HIF1α and that MITF co‐regulates a set of hypoxia response genes including *VEGFA* and *SLC5A9*. We show that phenotypically distinct melanoma cells lines exhibit largely different hypoxia responses, with only a few core genes being commonly regulated between them. This implies that in vivo different phenotypic subpopulations are likely to respond differently to the same microenvironmental cue. Since we also demonstrate that MITF regulates the TCA cycle to suppress pseudo‐hypoxia, the results provide a key insight into the role of MITF and hypoxia in phenotype switching.

## 1 INTRODUCTION

An increasingly recognized source of therapeutic resistance is provided by phenotypic heterogeneity whereby individual cells within tumors are exposed over time to different microenvironmental signals that drive them to adopt specific phenotypic states. At least four phenotypic states are likely to coexist within tumors that together can account for the biology of cancer progression: differentiated cells expressing cell‐type specific markers such as pigmentation genes in the case of melanoma; proliferating, KI‐67‐positive cells that frequently make up no more than 15%–20% of cells (Li, Jiang, Chen, & Zheng, 2015) but which are responsible for tumor expansion; invasive cells that seed metastases; and dormant cells that can give rise to relapse even many years after an apparent successful therapy (Sosa, Bragado, & Aguirre‐Ghiso, 2014). Since many therapies tend to target proliferating cells, cells exhibiting slow‐cycling properties are apt to exhibit resistance (Roesch et al., 2010, 2013), and recent gene expression profiling (Johannessen et al., 2013; Tsoi et al., 2018) and single‐cell RNA‐seq experiments (Jerby‐Arnon et al., 2018; Rambow et al., 2018; Tirosh et al., 2016) in melanoma suggest that resistance may be conferred by multiple phenotypic states bearing the same driver mutation.

Understanding the complex interplay between the microenvironment and cancer cells, and more specifically the molecular events underpinning the transition between phenotypic states, termed phenotype switching, can provide therapeutic opportunities. Unlike genetic lesions that are fixed, phenotype switching is dynamic and reversible and is therefore potentially amenable to therapies directed toward inducing cells to convert from drug‐resistant to drug‐sensitive states (Gupta et al., 2009; Saez‐Ayala et al., 2013). As such, understanding how microenvironmental cues drive cells toward specific phenotypes, and whether different phenotypic states exhibit specific therapeutic vulnerabilities is an important issue.

In addition to nutrient availability and signals from infiltrating immune cells and the stroma, one of the major intra‐tumor microenvironment signals is hypoxia. Tumor growth is associated with poorly organized vasculature leading to reduced oxygen delivery that can fail to meet the demands of tumor cells, and hypoxia is associated with worse prognosis (Bertout, Patel, & Simon, 2008). Hypoxia impacts metabolism (Marchiq & Pouyssegur, 2016), causing a switch away from oxidative phosphorylation toward glycolysis (Semenza, 2013), and can also promote metastasis (Semenza, 2012). In response to low oxygen, cells mount an adaptive response leading to the activation of a set of hypoxia‐inducible factors (HIFs) (Ivan et al., 2001; Jaakkola et al., 2001; Mahon, Hirota, & Semenza, 2001). The HIF transcription factors, HIF1α, HIF1β and HIF2α, then drive a program of gene expression directed toward mitigating the effects of hypoxia, including regulation of pH of the extracellular environment and promotion of de novo blood vessel growth (Marchiq & Pouyssegur, 2016; Semenza, 2013). However, increasing evidence appears to suggest that different cell types may exhibit specific hypoxia responses, since there appears to be little overlap (2%–28%) between genes differentially expressed under hypoxia in cells of different origins (Benita et al., 2009; Chi et al., 2006; Denko et al., 2003; Loftus et al., 2017; Widmer et al., 2013).

Melanoma, a highly aggressive skin cancer, represents an excellent opportunity to understand the impact of hypoxia on phenotypic heterogeneity. Much of melanoma biology is driven by the activity of the microphthalmia‐associated transcription factor MITF (Goding & Arnheiter, 2019) that promotes differentiation (Carreira et al., 2005) and proliferation (Du et al., 2004), and whose expression is anti‐correlated with invasion (Carreira et al., 2006) and therapy resistance (Dugo et al., 2015; Konieczkowski et al., 2014; Landsberg et al., 2012; Muller et al., 2014; Riesenberg et al., 2015; Tirosh et al., 2016). Indeed, in recognition of its key role in promoting melanomagenesis, MITF has been termed a lineage survival oncogene (Garraway et al., 2005). Given the importance of MITF in determining the phenotypic state of melanoma cells, there is considerable interest in understanding how it might be regulated by the intra‐tumor microenvironment. Consequently, several studies have examined the role of hypoxia in melanoma. Hypoxia leads to transcriptional silencing of MITF via an indirect mechanism involving HIF‐mediated up‐regulation of the transcription factor bHLHE40/DEC1 that then represses MITF expression (Cheli, Giuliano, et al., 2011a; Feige et al., 2011). Consistent with this, hypoxia can promote increased invasion and de‐differentiation in proliferative phenotype melanomas, but not those with a pre‐existing invasive phenotype (Widmer et al., 2013). Stabilization of HIF reportedly promotes invasion, with increased HIF‐dependent metastasis formation requiring platelet‐derived growth factor receptor alpha (PDGFRA) and focal adhesion kinase (FAK) (Hanna et al., 2013). Moreover, hypoxia has been suggested to drive a switch from proliferation‐associated receptor tyrosine kinase‐like orphan receptor 1 (ROR1) expression to invasion‐associated ROR2 expression (O'Connell et al., 2013).

Yet despite these advances, several key questions regarding the impact of hypoxia in melanoma remain. While previous studies have focused on a few selected genes (Widmer et al., 2013), or the hypoxia response in mouse melanocytes (Loftus et al., 2017), the impact of hypoxia on genomewide gene expression or which genes are direct targets of the HIF family in melanoma remains unknown. Nor is it clear whether melanomas exhibit a melanoma‐specific hypoxia signature compared to hypoxia in other cell types, whether some melanoma cells with distinct phenotypes exhibit a differential response to hypoxia, or whether MITF may contribute to the adaptive response to hypoxia. Neither is it known if a constitutive pseudo‐hypoxia gene expression signature found in some other cancer types under normoxic conditions exists in some melanomas.

## 2 MATERIALS AND METHODS

### 2.1 Cell lines

All cell lines were grown at 37°C with 10% CO_2_ in RPMI‐1640 (Gibco BRL, Invitrogen) supplemented with penicillin and streptomycin, 10% fetal bovine serum (FBS, Biosera). For starvation experiments dialyzed serum was used. The 501mel cells expressing the shRNA against HIF1α were constructed using HIF pGIPZ constructs (Open Biosystems/Thermo). The 501mel iMITF cell line was constructed as described previously (Falletta et al., 2017). All cell lines were tested monthly for mycoplasma and authenticated by Eurofins‐Genomics. siMITF (AGCAAGUACCUUUCUACCAC) (custom order, QIAGEN), sihsMITF#1 (UGGCUAUGCUUACGCUUAA), sihsMITF#3 (AGACGGAGCACACUUGUUA) (Dharmacon) transfections were carried out using Lipofectamine RNAiMax (Invitrogen #13778−150), according to the manufacturer's protocol.

### 2.2 Western blotting

Hot SDS–PAGE loading buffer (78.0 mM Tris [pH 6.8], 4% SDS, 20% glycerol, 0.2% bromophenol blue, supplemented with 100 mM DTT) was used to lyse cells before being subjected to SDS–PAGE using 12% acrylamide. Proteins transferred to nitrocellulose membranes (Amersham Biosciences) that were blocked with 5% non‐fat milk, in PBS containing 0.1% Tween‐20 before probing with primary antibodies (see below) overnight at 4°C. Proteins were detected using anti‐mouse, anti‐rabbit, or anti‐goat immunoglobulin coupled to horseradish peroxidase (Bio‐Rad, Santa Cruz) and visualized using an ECL detection kit (Amersham Biosciences) and X‐ray film (Fuji).

### 2.3 Invasiveness assays

Matrigel invasion assays were performed using an invasion chamber from BD Biocoat. Cells were seeded at 2 × 10^5^ per insert and cultured overnight in triplicate before treatment with DMOG. After 48‐hr incubation, cells remaining above the insert membrane were removed by gentle scraping with a sterile cotton swab. Cells that invaded through the Matrigel to the bottom of the insert were fixed in ethanol for 10 min, washed in PBS, and stained with methylene blue. The insert was then washed in PBS, air‐dried, and invading cells counted.

### 2.4 Antibodies

The primary antibodies used were as follows: mouse anti‐HIF1α (BD Biosciences), rabbit anti‐HIF1α (D2U3T), rabbit anti‐NDRG1 (D8G9), rabbit anti‐BNIP3 (D7U1T), rabbit anti‐ATF4 (D4B8), mouse anti‐LAMP1 (D4O1S) (Cell Signaling Technology), rabbit anti‐ERK2 (C14), mouse anti‐GAPDH (6C5), goat anti‐LDHA (N14) (Santa Cruz Biotechnologies), mouse anti‐MITF (C5) (Millipore), mouse anti‐MITF (D5) (Dako), rabbit anti‐MITF (HPA003259), rabbit anti‐SDHB (HPA002868) (Cambridge Biosciences). Rabbit anti‐HIF1α and anti‐HIF2α used in ChIP‐seq experiments were provided by the Ratcliffe laboratory, rabbit anti‐HIF1β (Novus Biologicals), mouse anti‐FLAG (M2) (Sigma), and mouse anti‐HA (12CA5) (Roche). Alexa Fluor‐conjugated secondary antibodies were obtained from Invitrogen.

### 2.5 Metabolomic analysis

Cells from 10 cm dishes were washed twice with 10 ml 5% mannitol in MilliQ water before metabolites were extracted from melanoma cell pellets using 1 ml methanol for 10 min and samples were deproteinized using 400 µl CHCl_3_ and 200 ml MilliQ water followed by centrifugation at 10,000 *g* for 3 min at 4°C. 400 µl of the aqueous layer was then filtered using a 5 kDa ultrafiltration tube and analysed by capillary electrophoresis mass spectrometry (CE‐MS) after addition of 25 µl 200 mM internal standards: L‐methionine sulfone (Wako 502–76641), 2‐(*N*‐morpholino) ethanesulfonic acid (Dojindo 349–01623), D‐Camphor‐10‐sulfonic acid (Wako 037–01032), 3‐aminopyrrolidine (Aldrich 404624) and trimesate (Wako 206–03641) as described (Kami et al., 2013) on an Agilent capillary electrophoresis system consisting of an Agilent G6220A LC/MSD TOF, an Agilent 1100 series isocratic HPLC pump, a G1603A Agilent CE‐MS adapter kit, and a G1607A Agilent CE‐ESI‐MS sprayer kit (Agilent Technologies, USA).

### 2.6 Succinate calorimetric assay

Intracellular succinate level was measured using Succinate Assay Kit (Colorimetric) (Abcam #ab204718) as per manufacturer instruction and normalized to cell counts as determined using TC20 automated cell counter (Bio‐Rad).

### 2.7 ChIP‐seq

Cells from three 80% confluent 15 cm dishes were trypsinized, collected into 50 ml falcon tube (Corning # 430,828), centrifuged (800 ×*g*, 4 min), and media aspirated. Cross‐linking was done by adding 35 ml ice‐cold PBS containing 0.4% paraformaldehyde. Cells were rotated at 4°C, 10 min before quenching with glycine to a final concentration of 0.2 M for 10 min. Samples were then washed and centrifuged (1,500 ×*g*, 10 min). Lysis was done in 1 ml ChIP lysis buffer (50 mM Tris–HCl (pH 8.0), 10 mM EDTA, 10 mM sodium butyrate, 1% SDS, 0.5 mM PMSF, 4 × PIC (Roche #05056489001) by passing the cell suspension through a 25‐gauge needle until there were no visible clumps. A further 1 ml of ChIP dilution buffer was added before sonication for ≈12 min in a Covaris S220 (160 W peak incident power, 5% duty cycle, and 200 cycles per burst) until 200–400 bp fragments were obtained (assessed by agarose gel electrophoresis). The sonicated chromatin was cleared by centrifugation at 13,000 ×*g*, 10 min and the supernatant diluted in 8 ml of ChIP dilution buffer (10 ml total, 1.67 mM Tris (pH 8.0), 167 mM NaCl, 1.2 mM EDTA, 1% Triton X‐100, 0.01% SDS) before 80 µg of respective antibodies was added and chromatin rotated in a 50‐ml falcon tube overnight. In parallel, 400 µl Dynabeads Protein A or G were washed, resuspended in ChIP dilution buffer, and blocked in 0.5 mg/ml BSA overnight. Immunoprecipitation was carried out using blocked Dynabeads, rotated for 1 hr, and centrifuged (1,500 ×*g*, 10 min). The beads were resuspended in 1 ml ChIP low salt wash buffer (20 mM Tris–HCl (pH 8.0), 150 mM NaCl, 2 mM EDTA, 1% Triton X‐100, 0.1% SDS) and transferred to a fresh microcentrifuge tube, washed, and resuspended in a further 1 ml ChIP low salt wash buffer before transferring to a fresh microcentrifuge tube. Further washing, 2× ChIP low salt wash buffer, 2× ChIP high salt wash buffer (20 mM Tris–HCl (pH 8.0), 500 mM NaCl, 2 mM EDTA, 1% Triton X‐100, 0.1% SDS) and 2× LiCl wash buffer (10 mM Tris–HCl (pH 8.0), 250 mM LiCl, 1 mM EDTA, 1% sodium deoxycholate, 1% NP–40) was done in the same tube. The beads were eluted in 1.2 ml elution buffer (100 mM NaHCO_3_, 1% SDS). Reverse cross‐linking of ChIPed‐DNA was done at 65°C overnight with addition of 0.3 M NaCl (final concentration), 20 µg RNase A, and 20 µg Proteinase K. Recovery of ChIPed‐DNA was done using QIAquick PCR Purification Kit. 6 ml of PB buffer was added to the reversed cross‐linked DNA before passing through four columns as described per supplier's instruction. Final elution was done by passing 30 µl water sequentially through all the columns. This fraction was kept for ChIP‐seq library preparation, 1 µl of which was assessed on a Bioanalyzer. Samples which passed the QC on the Bioanalyzer (fragment length distribution primarily around 200‐400bp) and showed enrichment at expected targets on qPCR were subjected to sequencing on HiSeq 2500 (Illumina) carried out using the Wellcome Trust genomic service, Oxford.

### 2.8 RNA‐seq

RNA was extracted using RNeasy kit (QIAGEN #74106), and QC on the Bioanalyzer (for RIN ≥9.5). ERCC ExFold RNA Spike‐In Mixes (Ambion) was added prior to Library prep using QuantSeq Forward kit (LEXOGEN #0.15.96), using 500 ng starting material to minimize the PCR amplification step. Samples were sequenced on HiSeq 4000 (Illumina) carried out using the Wellcome Trust genomic service, Oxford.

### 2.9 Bioinformatics for ChIP‐seq

Each replicate contained two technical replicates (same library sequence in two separate flow cells) which were stitched together using UNIX to generate a single fastq. Raw fastq files were fastQCed to check the read quality and PCR duplication, processed, and mapped to human genome build hg19 (GRCh37, February 2009) using Bowtie (Langmead & Salzberg, 2012; Langmead, Trapnell, Pop, & Salzberg, 2009) allowing for 2 mismatches. Mapped SAM‐files were used for peak calling using the Homer package (Heinz et al., 2010). The background files used were generated by performing a parallel ChIP‐seq experiment using HA antibody against 501mel parental cell lines. Peak annotation, genome ontology analysis, de novo motif identification, and bedgraph generation were carried out using the Homer package with peaks being assigned to the nearest gene. Peaks were further filtered for those with peak score <10 to increase stringency of the analysis. Peaks were identified as co‐occupying a genomic location if the peak summit or the start coordinate of the peak lay within 200 bp.

### 2.10 Bioinformatics for RNA‐seq

Fastq files were treated as for ChIP‐seq. Raw fastq files were then trimmed of poly‐A using cutadapt (Martin, 2011) and mapped using STAR (Dobin et al., 2013) against hg38 (GRCh38, 2015). Counts per gene from STAR were used as input for differential gene expression analysis using EdgeR (Robinson, McCarthy, & Smyth, 2010). Reads for each sample set were first filtered for genes whose expression is <1 count per million prior to glmQLFTest. Genes with a *p *≤ 0.05 and fold‐changes above 2 were taken for further analysis. In the case of shHIF1α, genes whose differential gene expression was dampened by ≥8‐fold were taken as significant. Heatmaps of RNA‐seq samples were generated from the edgeR‐library normalized reads of genes whose differential gene expression has *p *≤ 0.05 and fold change ≥2 before center normalized and cluster using ComplexHeatmaps (Gu, Eils, & Schlesner, 2016).

### 2.11 GSEA and GSVA analyses

GSEA analyses were carried out using javaGSEA2‐3.0 (Subramanian et al., 2005). 10,000 permutations were carried out for each probed gene set. Maximum gene set size was set to 800 to accommodate the Verfaillie invasive gene set. GSVA analyses were performed using the Bioconductor package GSVA (Hänzelman, Castelo, & Guinney, 2013). The gene sets used were obtained from the Molecular Signatures Database (Subramanian et al., 2005). The GSVA matrix was then clustered and displayed as heatmap using Pheatmap (https://cran.r-project.org/web/packages/pheatmap/index.html).

TCGA Expression data were retrieved using CGDS‐R package (https://cran.r-project.org/web/packages/cgdsr/index.html).

## 3 RESULTS

### 3.1 Hypoxia in melanoma correlates with invasiveness

Previous work has established an inverse correlation between hypoxia and expression of differentiation markers in melanoma in a restricted set of melanoma tissue sections and has suggested that hypoxia may induce de‐differentiation and invasiveness in melanoma or in melanocytes (Cheli, Giuliano, et al., 2011a; Loftus et al., 2017; Widmer et al., 2013). However, whether hypoxia correlates with invasion in melanoma in general has not been examined in detail. We therefore analyzed the TCGA melanoma cohort for correlations with the previously published Elvidge hypoxia gene expression signature comprising 171 genes that are up‐regulated in response to hypoxia (Elvidge et al., 2006). Each melanoma was ranked by a score corresponding to the average expression of the genes in the Elvidge hypoxia gene set. We then examined each individual melanoma for the expression of the Verfaillie melanoma invasive gene expression signature (Verfaillie et al., 2015). While there was some variation in the invasive gene expression signature score between individual melanomas, a moving average of the Verfaillie signature showed a high degree of correlation with the hypoxic signature (Figure S1a). The noise in the moving average was largely abolished when we compared the Verfaillie invasive signature to the Elvidge hypoxia signature in a single‐cell RNA‐seq analysis (Tirosh et al., 2016) of melanoma cells from dissociated tumors (Figure S1b), suggesting the noise may arise from non‐melanoma cells within the TCGA melanoma samples. Note that only 31 genes are found in both gene sets, and these do not account for the correlation between the hypoxia and invasive signatures. The hypoxia signature was also strongly inversely correlated to MITF expression (Figure S1c), consistent with hypoxia repressing MITF to drive a de‐differentiated phenotype (Cheli, Giuliano, et al., 2011a), and correlated strongly with the expression of *AXL* (Figure S1d) encoding a receptor tyrosine kinase linked to an MITF‐low, AXL‐high drug resistance phenotype (Dugo et al., 2015; Konieczkowski et al., 2014; Muller et al., 2014). Gene set enrichment analysis (GSEA) of the top and bottom 75 TCGA melanomas ranked by the Elvidge hypoxia gene expression signature also confirmed a strong enrichment in the top 75 hypoxic melanomas for the Verfaillie invasive gene set (Figure S1e) and epithelial–mesenchyme transition (EMT)‐associated genes (HALLMARK EMT) (Figure S1f). As expected, given the inverse correlation in melanoma between proliferation and invasion (Carreira et al., 2006), the 75 TCGA melanomas exhibiting the highest hypoxic gene expression exhibited a reduced proliferative gene expression signature (Verfaillie et al., 2015) compared to the bottom 75 (Figure S1g). That hypoxia could induce invasion was confirmed using DMOG, a cell‐permeable prolyl‐4‐hydroxylase inhibitor, to impose a hypoxia gene expression program. As anticipated, DMOG transiently induced HIF1α expression and increased invasiveness in both IGR37 and 501mel human BRAF^V600E^‐mutated melanoma cell lines (Figure S1h).

Hypoxia should reduce oxidative phosphorylation that occurs in mitochondria (Semenza, 2013), and hypoxia‐mediated suppression of MITF that controls expression of PPAR gamma cofactor 1 alpha (PGC1α; PPARGC1A), a key factor implicated in mitobiogenesis, would also contribute to an altered metabolic state. Consistent with this, comparison between the top and bottom 75 TCGA melanomas ranked by the Elvidge hypoxia signature using gene set variation analysis (GSVA) revealed a strong down‐regulation of a mitobiogenesis signature (Figure S1i) previously associated with BRAF inhibitor resistance (Zhang et al., 2016). This was also apparent in the melanoma cell lines in the Cancer Cell Line Encyclopedia where the mitobiogenesis signature was used to interrogate the top and bottom 20 lines ranked by the Elvidge hypoxia signature. A clear subset of the top 20 melanoma cell lines exhibiting a constitutive hypoxic signature, termed pseudo‐hypoxia, under normoxic culture conditions showed a strong down‐regulation of the mitobiogenesis signature (Figure S1j).

Recent advances in melanoma therapy have seen a shift away from BRAF targeted therapies toward those aimed at reactivating the immune system. However, as resistance to immune checkpoint therapies is frequently encountered, we asked whether the Elvidge hypoxia signature would also correlate with a recently characterized gene expression signature that correlates with innate anti‐PD‐1 resistance (IPRES) (Hugo et al., 2016). Strikingly, GSVA of the top 75 TCGA melanomas ranked by the Elvidge hypoxia signature showed they were very strongly enriched for the IPRES signature (Figure S1k), as were a subset of the CCLE melanoma cell lines ranked by the Elvidge hypoxia signature (Figure S1l). Collectively these analyses indicate that in melanomas, hypoxia correlates with invasion, drug, and immune checkpoint inhibitor resistance and negatively correlates with mitobiogenesis, differentiation, and proliferation. They also indicate that a subset of melanoma cell lines exhibit a constitutive pseudo‐hypoxia gene expression signature even when grown under normoxic conditions.

### 3.2 Identification of a core hypoxic response signature between melanoma cell lines

Although these data provide an indication of the how tumors respond to hypoxia, the microenvironment within tumors is highly complex and it is possible that additional signals within the hypoxic microenvironment could contribute to the correlations observed. Moreover, it is unclear whether all melanoma cells will exhibit a common hypoxia response, or whether different phenotypic subpopulations of cells within a tumor will mount a different hypoxia response. To address these issues, we examined the gene expression signature of three different BRAF^V600E^ mutant melanoma cell lines in response to hypoxia over time in biological triplicate using a 3’RNA‐seq approach. The cell lines used were IGR39, that is MITF‐low, highly de‐differentiated, invasive, and drug‐resistant (Konieczkowski et al., 2014); IGR37 that is MITF‐positive, non‐invasive, and isolated from the same patient as IGR39 (Luis et al., 1989); and 501mel (Shamamian et al., 1994), that expresses high levels of MITF, is non‐invasive, and was isolated from a different patient than the IGR37 and IGR39 cell lines. Importantly, rather than examining gene expression at a single 24 hr time point, as has been done previously for a mouse melanocyte cell line (Loftus et al., 2017), we chose to assess the effects of hypoxia over time. This is because the stabilization of HIF1α in response to low oxygen tends to be short‐lived while HIF2α mediates a longer term response (Koh & Powis, 2012) and we wished to capture both short‐ and long‐term effects on gene expression as well as any dynamic changes.

**Figure 1:**
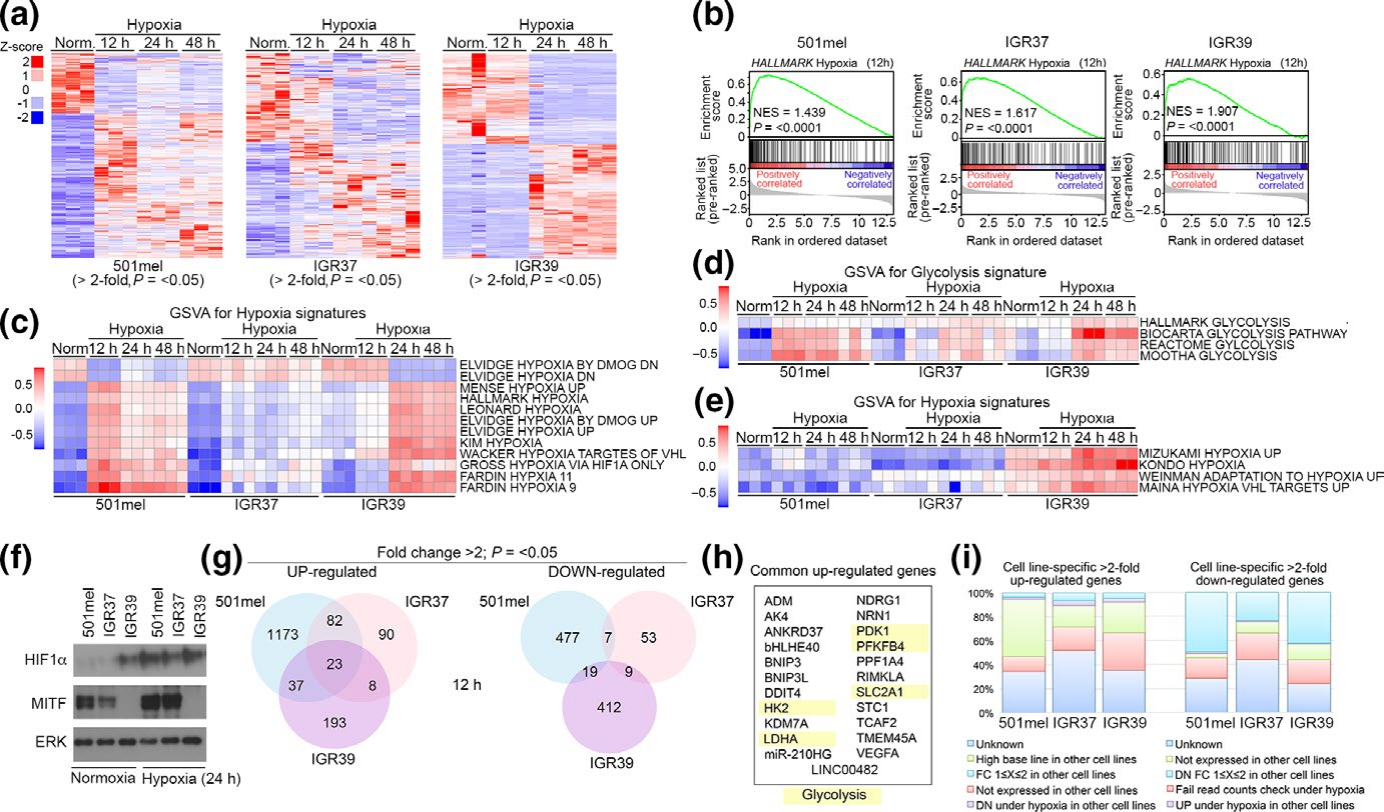
Identification of a common set of melanoma hypoxia‐regulated genes. (a) Heatmaps derived from triplicate RNA‐seq analysis showing differential gene expression in three melanoma cell lines grown in normoxia or in 1% oxygen for indicated times. Only those genes differentially regulated by more than twofold and *p =* < 0.05 are shown. (b) GSEA analyses showing enrichment of the Elvidge hypoxia gene expression signature in indicated cell lines grown in normoxia or 1% oxygen for 12 hr. (c–e) Heatmaps showing results of GSVA for indicated gene sets for three melanoma cell lines grown in normoxia or 1% oxygen for indicated times. (f) Western blot of indicated cell lines grown in normoxia or hypoxia for 24 hr. (g) Venn diagrams showing unique and co‐regulated genes up‐ or down‐regulated by at least twofold (*p* < 0.05) in response to 1% oxygen in the three melanoma cell lines after 12 hr in hypoxia. (h) List of genes commonly up‐regulated in response to hypoxia in the three melanoma cell lines examined after 12 hr in hypoxia. (i) Bar charts indicating potential reasons why few genes in common are regulated by hypoxia 12 hr after exposure to 1% oxygen in all three cell lines

Heatmaps highlighting the gene expression programs in normoxia versus hypoxia in the three cell lines over time are shown in Figure 1a and a list of regulated genes for each cell line presented in Table S1. GSEA analysis confirmed that under hypoxia all three cell lines exhibited enrichment for the HALLMARK_HYPOXIA gene set (Figure 1b). GSVA confirmed these observations and showed that several previously established hypoxia response gene sets were enriched in the melanoma cell lines under hypoxia conditions (Figure 1c), though enrichment was less robust in the IGR37 cells.

Hypoxia, and specifically HIF1α, imposes a metabolic shift away from oxidative phosphorylation and toward glycolysis (Semenza, 2013). Consistent with this, all three lines exhibited enrichment in expression of gene sets associated with glycolysis (Figure 1d). We were especially interested in any difference in the response between the differentiated IGR37 and undifferentiated IGR39 cell lines. In normoxic conditions, these two cell lines possess substantially different gene expression programs (Figure S2a). GSVA of several EMT‐associated gene expression signatures revealed that IGR39 cells were enriched for EMT gene expression (Figure S2b) even under normoxia. This is consistent with the IGR39 cell line having an MITF‐low, invasive phenotype. No significant enrichment of EMT signatures was observed for the IGR37 or 501mel lines under hypoxia at the 12 hr time point. Significantly, IGR39 cells, but not IGR37 or 501mel cells, also exhibited enrichment of a panel of hypoxia‐associated gene sets even under normoxic conditions (Figure 1e). Western blotting revealed that both NDRG and BNIP3, well‐characterized hypoxia‐induced HIF targets, were constitutively expressed in IGR39 cells compared to the 501mel and IGR37 cell lines (Figure S2c), though BNIP3 was nicely inducible using DMOG in 501mel and IGR37 cells (Figure S2d). Consistent with these observations, the MITF‐negative IGR39 cell line constitutively expressed HIF1α (Figure 1f). The IGR39 cell line may therefore be representative of the subset of constitutively “hypoxic” melanoma lines identified in the CCLE collection (Figure S1j, l). In agreement, GSVA revealed IGR39 cells expressed a constitutive IPRES signature compared to IGR37 cells (Figure S2e), though this was enhanced under hypoxic conditions (Figure S2f). Note that in contrast to previous reports that showed MITF is down‐regulated in hypoxia (Cheli, Giuliano, et al., 2011a; Feige et al., 2011), we observed MITF protein levels were increased after 24 hr exposure to hypoxia in the MITF‐positive 501mel and IGR37 cell lines (Figure 1f) raising the possibility that short‐term hypoxia can up‐regulate MITF expression (see also below).

We next examined whether the three melanoma cell lines mounted a similar or different response to hypoxia. Using a statistical cutoff corresponding to twofold induction under hypoxia and a *p *< 0.05, Figure 1g shows the number of up‐ or down‐regulated genes in each cell line at 12 hr, with the 24 hr and 48 hr time points shown in Figure S2g. Notably each cell line exhibited a substantial number of genes whose induction under hypoxia was unique to that cell line, and each pair of cell lines also exhibited an overlap that was not apparent with the third cell line. Only 23 genes were up‐regulated in common between the three cell lines under hypoxia at the 12 hr time point, including one long non‐coding RNA (Figure 1h; Table S2), and no genes were down‐regulated in all three cell lines. At 24 or 48 hr, the number of commonly regulated genes was increased (Figure S2g), but by this time, indirect effects on gene expression are more likely.

Since so few genes were up‐ or down‐regulated in common between the three cell lines, we explored some potentially contributing factors that might explain why different melanoma cell lines have such different responses to hypoxia (Figure 1i). In 501mel cells, we found a significant proportion of the uniquely up‐regulated genes in this line were not expressed in the other two cell lines, perhaps because of epigenetic silencing that would prevent activation by hypoxia. Moreover, almost 50% of the hypoxia‐responsive genes also had high basal level expression in the other cell lines; it may be more difficult for the hypoxia‐inducible transcription factors to amplify expression of a gene that is already being highly transcribed. While a reduced fold activation in the other cell lines, below our statistical cutoff, could also account for a proportion of “unique” to 501mel response genes, around 35% remained unexplained. Similar observations were made for the uniquely responsive genes in the other cell lines.

The common up‐regulated genes (Figure 1h) included some implicated in several key steps in glucose uptake (*SLC2A1*), glycolysis (*HK2, PFKFB4, PDK1*) and the pyruvate–lactate axis (*LDHA*), as well as genes implicated in epigenetic control (*KDM7A*) and angiogenesis (*VEGFA*). Thus, after 12‐hr exposure to hypoxia, all three melanoma cell lines had already reprogrammed their transcription toward glycolytic metabolism. Also, significant was up‐regulation of *BHLHE40/DEC1*, a transcription factor reported to mediate transcriptional repression of MITF under hypoxia (Cheli, Giuliano, et al., 2011a; Feige et al., 2011).

Interestingly, no genes implicated in invasion or EMT were commonly up‐regulated, and although DMOG could trigger invasion (Figure S1h), we were unable to increase invasiveness in the melanoma cell lines grown in 1% oxygen (not shown). This result was unexpected because hypoxia is known to induce metastatic spread and EMT signatures are enriched in hypoxic melanoma tumors (Figure S1a,b,e,f). This may be because we assayed for gene expression changes at 1% oxygen, which is sufficient to induce HIF1α and reprogramming of metabolic gene expression, but lower oxygen levels found within tumors and mimicked by DMOG may be necessary to promote invasion.

### 3.3 HIF target genes in melanoma

**Figure 2:**
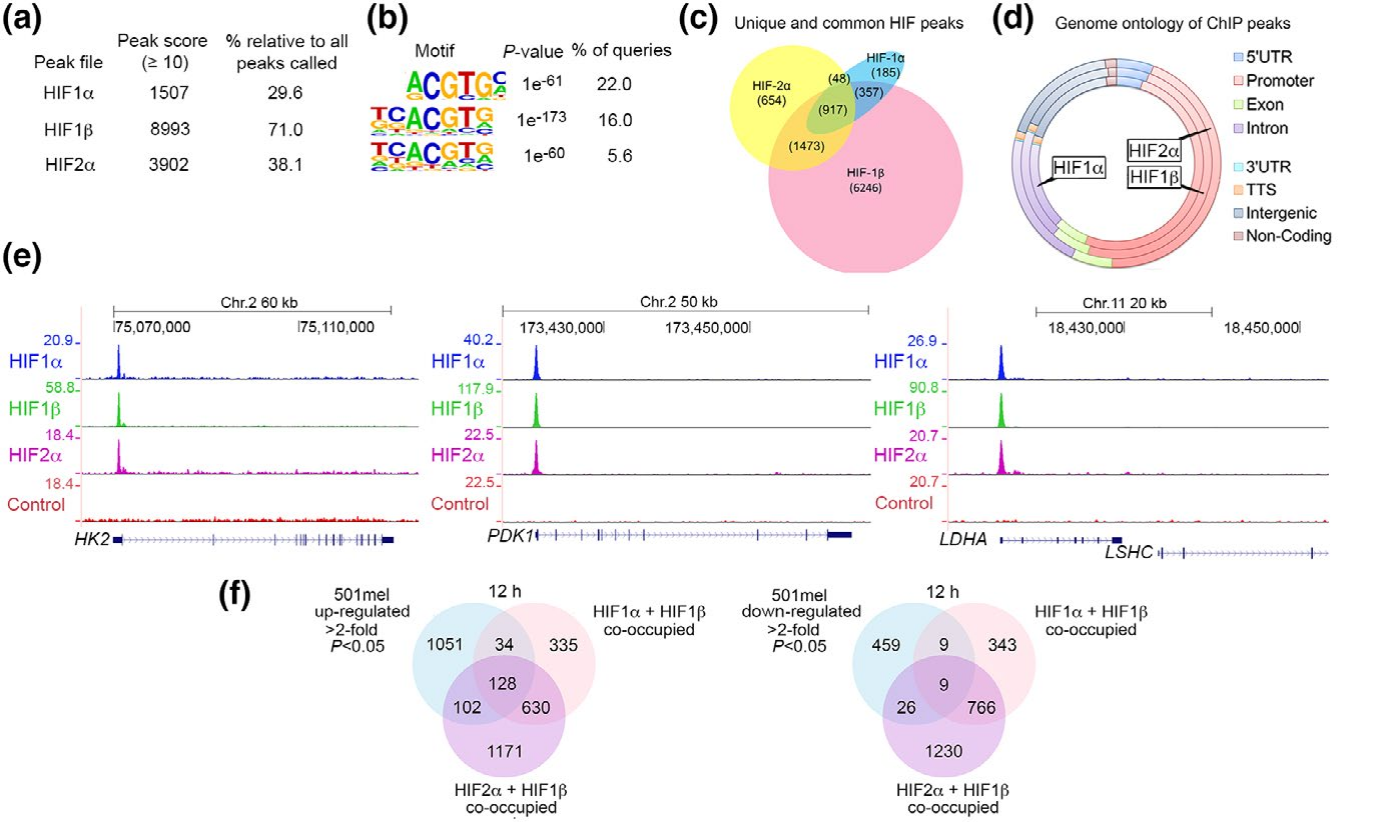
Genomewide binding of the HIFs in 501mel cells (a) Number of binding sites with peak score above 10 for each of the three HIFs derived from the ChIP‐seq analysis in the 501mel cell line. (b) Consensus motifs for each of the three HIFs determined from the ChIP‐seq analysis. (c) Venn diagram showing common and uniquely occupied sites. (d) Genome ontology of HIF binding sites. (e) UCSC genome browser screenshots of occupied sites in the 501mel cell line for three of the genes commonly regulated in the three melanoma cell lines. (f) Venn diagrams integrating ChIP‐seq and RNA‐seq datasets from 501mel cells for genes bound by the HIFs and twofold up‐ or down‐regulated at 12 hr post‐hypoxia as indicated

To identify which genes might be direct HIF targets, we examined the genomewide binding of each of the three hypoxic response transcription factors, HIF1α, HIF1β and HIF2α in the 501mel cell line using chromatin immunoprecipitation followed by high‐throughput sequencing (ChIP‐seq). In all, 1,507, 8,993, and 3,092 sites were bound by HIF1α, HIF1β, and HIF2α respectively (Figure 2a) and a complete list of binding sites is provided in Table S3. The recognition motif for each factor derived from the ChIP‐seq analysis (Figure 2b) reflected the known consensus, ACGTG, though for both HIF2α and HIF1β, the consensus motif was extended to CACGTG, an E‐box of which around 50% possessed a 5′ T flanking residue, a hallmark of MITF binding sites (Aksan & Goding, 1998). Of the HIF binding sites detected, 1,473 were bound by both HIF1β and HIF2α, while 357 were co‐occupied by HIF1α and HIF1β (Figure 2c). Only 48 exhibited co‐occupancy by HIF1α and HIF2α alone, but 917 were bound by all three factors. Since the HIF family binds DNA as heterodimers, the detection of all three would suggest exchange of dimers at the same location. A similar genomic distribution was observed for each HIF family member (Figure 2d), with promoter binding being the most prevalent location within bound genes. Examples of loci co‐bound by all three factors and corresponding to genes commonly regulated by hypoxia in all three melanoma cell lines examined (*HK2*, *PDK1,* and *LDHA*) are shown in Figure 2e.

Integration of the 501mel ChIP‐seq data with the corresponding gene expression RNA‐seq dataset revealed that of the genes up‐regulated in response to 12 hr hypoxia, 1,051 (80%) were not bound by either HIF1α/HIF1β or HIF2α/HIF1β and were therefore likely regulated indirectly, 128 were bound by both heterodimers, with 34 and 102 genes bound by HIF1α/HIF1β or HIF2α/HIF1β, respectively (Figure 2f). However, a majority of the genes bound by the HIFs did not exhibit significant levels of up‐regulation in response to 12 hr hypoxia. For the down‐regulated genes, 459 were regulated indirectly, and only 44 (9%) in total were bound by any combination of HIF1α/HIF1β or HIF2α/HIF1β (Figure 2f). Results for 24 and 48 hr post‐exposure to hypoxia are shown in Figure S3. In contrast to the up‐regulated genes therefore, very few hypoxia‐repressed genes are bound by the HIF factors and the majority of repression is likely to be indirect. Details of binding to the commonly up‐regulated genes identified are highlighted in Table S4.

### 3.4 MITF is transiently up‐regulated by HIF1α

**Figure 3:**
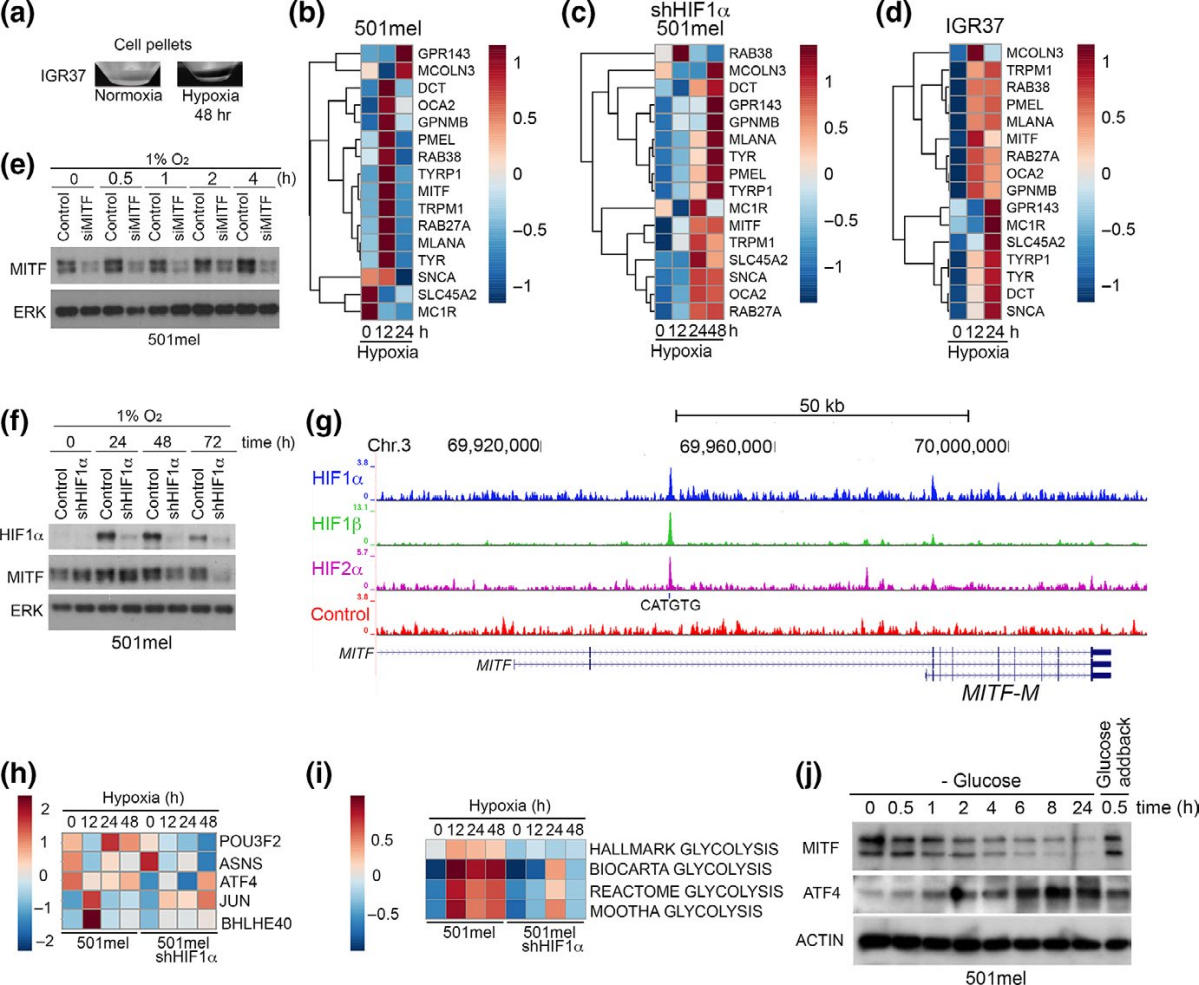
MITF is positively regulated by HIF1α. (a) Cell pellet from IGR37 cells grown in normoxia or hypoxia as indicated. (b–d) Heatmaps derived from triplicate RNA‐seq analysis showing relative expression of indicated genes from 501mel, 501mel‐shHIF1α, and IGR37 cells in normoxia or after indicated times in hypoxia. (e) Western blot of 501mel cells grown for indicated times in normoxia or hypoxia treated with control or siRNA specific for MITF. (f) Western blot of 501mel cells or 501mel‐shHIF1α cells grown for indicated times in 1% oxygen. (g) UCSC genome browser screenshot of HIF ChIP‐seq showing all three HIFs bound upstream from the MITF‐M promoter. (h,i) Heatmaps showing relative expression of indicated genes or gene sets from 501mel, 501mel‐shHIF1α cell lines grown in normoxia or after indicated times in hypoxia. (j) Western blot of extracts from 501mel cells grown in RPMI (15 mM glucose) or in medium lacking glucose (‐glucose) for indicated times, or after re‐addition of 15 mM glucose for 30 min as indicated

The results so far indicate that different melanomas cell lines exhibit a distinct hypoxia response, but share the key elements of metabolic reprogramming known to occur in other cell types in low oxygen conditions. However, we noted that in some experiments after 48 hr in hypoxia the IGR37 cell line, though not the MITF‐negative cell line IGR39, or 501mel, exhibited increased pigmentation (Figure 3a). Pigmentation is a differentiation function of melanocytes in which the production of the melanin is a consequence of the activity of a set of pigmentation enzymes, including tyrosinase (TYR), tyrosinase‐related protein 1 (TYRP1), and dopachrome tautomerase (DCT) within specialized organelles termed melanosomes (Park & Gilchrest, 1999). MITF coordinates melanin production by directly regulating most, if not all, genes implicated in melanin synthesis, and melanosome genesis (e.g., *PMEL* and *MLANA)* and transport (e.g., *RAB27A*) (Goding & Arnheiter, 2019). The increase in pigmentation in the IGR37 cells under hypoxia was therefore surprising since it is well established that the regulator of melanoma/melanocyte differentiation, MITF, is down‐regulated in hypoxia (Cheli, Giuliano, et al., 2011a; Feige et al., 2011). However, we had already observed that MITF is up‐regulated in both the IGR37 and 501mel cell lines 24 hr post‐hypoxia (Figure 1f). We therefore re‐examined the expression of MITF and several of its pigmentation‐associated target genes over time. Strikingly, in 501mel cell mRNA for MITF and many of its downstream differentiation targets were transiently up‐regulated at 12 hr following exposure to hypoxia, but expression was returned to baseline or below by 24 hr (Figure 3b). Using a 501mel cell line expressing an shRNA targeting HIF1α, the up‐regulation of MITF and its target genes also occurred in low oxygen conditions, but was severely delayed (Figure 3c). IGR37 cells, which undergo a less robust hypoxic response than 501mel cells (Figure 1c), also up‐regulated MITF and its target genes (Figure 3d), though with a delayed kinetic compared to the 501mel cells. The increased mRNA expression of MITF and its target genes in 501mel cells detected using RNA‐seq was reflected in a moderate up‐regulation of MITF protein within 4‐hr exposure to hypoxia (Figure 3e). Depletion of MITF using siRNA confirmed that the detected band was indeed MITF.

The unexpected observation that hypoxia could transiently increase MITF led us to ask whether inhibiting HIF1α would affect MITF expression over time. Using shHIF1α‐expressing 501mel cells, the induction of HIF1α in response to hypoxia was severely blunted as expected (Figure 3f), and while MITF expression was increased at 24 hr, its expression declined more rapidly in cells expressing shHIF1α. This suggested that HIF1α might positively regulate MITF expression. Consistent with this, the ChIP‐seq analysis revealed a previously uncharacterized binding site for all three HIF factors around 45 kb upstream from the promoter driving expression of the melanocyte/melanoma‐specific MITF‐M isoform (Figure 3g). Together with the RNA‐seq analysis (Figure 3b,c), this result is consistent with hypoxia transiently up‐regulating MITF and its downstream target genes via HIF1α binding.

We next asked why shHIF1α led to greater reduction in MITF levels at later times than was observed in control cells. Previous studies have identified bHLHE40/DEC1, a transcription factor directly up‐regulated by HIF1α in hypoxia as a direct repressor of MITF (Cheli, Giuliano, et al., 2011a; Feige et al., 2011). However, our observation that shRNA knockdown of HIF1α led to enhanced silencing of MITF suggested a mechanism for MITF repression that was bHLHE40/DEC1‐independent. Consistent with this, while bHLHE40/DEC1 mRNA expression was increased at 12 hr post‐hypoxia in 501mel cells, its up‐regulation was blunted in cells expressing shHIF1α (Figure 3h). Similarly, other known MITF transcription repressors POU3F2 (BRN2) (Goodall et al., 2008), JUN (Riesenberg et al., 2015), and ATF4 (Falletta et al., 2017; Ferguson, Smith, Zudaire, Wellbrock, & Arozarena, 2017) were also examined. Because ATF4 is primarily regulated by translation, we also included its downstream target ASNS. However, of the set of potential MITF repressors only JUN exhibited any slight increase in the shHIF1α cells on exposure to hypoxia.

Since hypoxia promotes increased expression of genes implicated in glycolysis and shHIF1α prevented the increased expression of glycolysis gene sets (Figure 3i), we hypothesized that under hypoxia MITF expression might be sustained by increased glucose uptake, consistent with low glucose suppressing MITF expression (Ferguson et al., 2017). In 501mel cells, glucose deprivation led to a progressive decrease in MITF expression (Figure 3j) accompanied by up‐regulation of ATF4, a transcriptional repressor of MITF (Falletta et al., 2017; Ferguson et al., 2017) that is induced under conditions of translation stress (Harding et al., 2000). This is reminiscent of the decrease in MITF mediated by translational reprogramming mediated by eIF2α phosphorylation observed on glutamine limitation (Falletta et al., 2017). Since eIF2α phosphorylation is a key determinant of melanoma phenotype (Falletta et al., 2017; Maida et al., 2019), our observations are consistent with elevated glucose uptake under hypoxia contributing to the maintenance of MITF expression.

### 3.5 MITF regulates a cohort of hypoxic response genes

**Figure 4:**
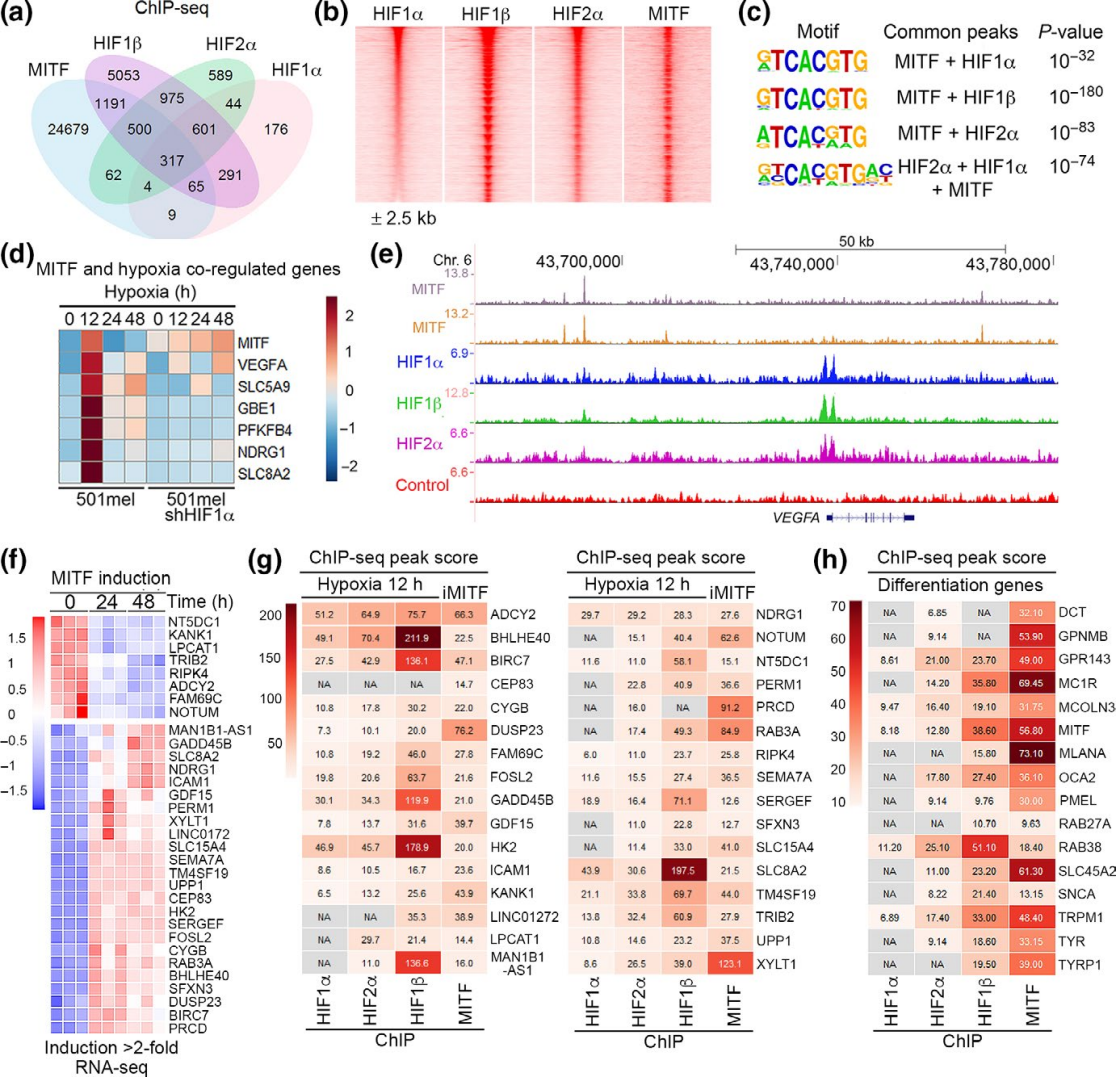
MITF regulates a subset of hypoxia‐responsive genes (a) Venn diagram showing ChIP‐seq peaks for each of the three HIF factors and MITF. Numbers indicated number of unique and overlapping peaks for each combination of experiment. (b) Read density maps of the ChIP‐seq profile for each HIF and MITF ranked by peak score for HIF1α centered on the co‐occupied sites ± 2.5 kb. (c) De novo motifs derived from the ChIP‐seq analysis of overlapping sites bound by each of the hypoxia factors as well as MITF. (d) Heatmap derived from triplicate RNA‐seq of 501mel cells or a derivative line expressing shHIF1α showing a set of genes bound and co‐regulated by HIF and MITF. (e) UCSC genome browser screenshot of HIF ChIP‐seq and a biological replicate of an MITF ChIP‐seq showing all three HIFs and MITF bound upstream from the *VEGFA* gene. (f) Heatmap derived from triplicate RNA‐seq showing differential expression of genes associated with peaks co‐occupied by HIFs and MITF in 501mel expressing doxycycline‐inducible MITF over time following induction. (g, h) Heatmap showing peak scores for the HIF family members and MITF on the indicated genes after 12 hr in 1% Oxygen

The fact that MITF and its target genes are transiently up‐regulated by hypoxia led us to ask whether MITF could co‐regulate a set of HIF targets. Given the related consensus motifs for DNA binding by MITF (CACGTG) and by HIF (ACGTG) (Figure 2b), it seemed likely that at least some genes would be co‐bound and co‐regulated. Using a threshold for the ChIP‐seq analysis of a peak score above 10, a total of 882 MITF target sites were also recognized by a HIF1α/HIF2α‐HIF1β combination (Figure 4a). Of these, 317 sites were co‐occupied by all four transcription factors, while 500 sites are unique to MITF‐HIF2α‐HIF1β and 65 are specific to MITF‐HIF1α‐1β. Examining the read densities from the ChIP‐seq analysis of all four factors ranked by peak score of HIF1α (Figure 4b) revealed extensive co‐occupancy at bound sites, though it was not likely that MITF would bind to sites simultaneously with the HIF factors. As might be expected, given the extended sequence requirement for MITF DNA binding (Aksan & Goding, 1998) compared to HIF, the consensus at the co‐bound sites match that of classical MITF TCACGTG targets rather than the shorter ACGTG hypoxia response element (Figure 4c). Some examples of well‐characterized hypoxia response genes co‐regulated by MITF are highlighted in Figure 4d*,* with their induction in response to inducible MITF shown in Table 1. For example, MITF and HIF binding to distinct sites in the *VEGFA* gene is shown in Figure 4e, and other examples of co‐bound and regulated genes are shown in Figure S4a. Notable among the co‐regulated genes are *VEGFA* that stimulates angiogenesis and the glucose transporter *SLC5A9*, consistent with MITF promoting glucose uptake like HIF1α (Figure 4d; Table 1). Some genes robustly up‐regulated by hypoxia and sharing a common binding site for MITF and HIF were also up‐regulated by MITF. Using a previously described (Falletta et al., 2017) 501mel cell line engineered to express doxycycline‐inducible MITF, we examined using RNA‐seq whether induction of MITF would regulate any of the genes regulated by hypoxia and bound by the HIFs. Examples are shown in Figure 4f, where induction of MITF over time leads to repression or activation of a set of hypoxia response genes. Examination of *NDRG1*, where the HIFs bind upstream from the gene, but MITF binds within an intron (Figure S4a lower panel), indicated that siRNA‐mediated depletion of MITF moderately enhanced the induction of *NDRG1* by DMOG in 501mel cells, but had no discernible effect in IGR37 cells (Figure S4b).

**Table 1:** Triplicate RNA‐seq data from a 501mel cell line inducibly expressing murine FLAG‐tagged MITF (501mel iMITF) in response to 100 ng doxycycline for 24 or 48 hr. Fold change in mRNA levels from indicated genes is presented relative to expression in uninduced cells

Gene	Fold change (24 hr)	Fold change (48 hr)	*p*	FDR	Gene function
MITF	0.11	0.11	3.55E−13	4.77E−11	Endogenous human MITF
PFKFB4	2.17	1.26	1.18E−04	2.37E−04	6‐Phosphofructo−2‐kinase
NDRG1	2.47	3.64	6.20E−09	5.50E−08	Stress–response tumor suppressor
SLC8A2	3.31	4.04	1.41E−06	4.79E−06	Na^+^/Ca_2_^+^ antiporter
SLC5A9	8.63	7.99	1.04E−09	1.43E−08	Glucose transporter
GBE1	8.82	9.63	9.18E−14	2.50E−11	Glycogen branching enzyme
VEGFA	3.48	2.68	3.34E−10	6.05E−09	Inducer of angiogenesis
Mitf‐FLAG	18.62	19.56	1.64E−10	3.69E−09	Ectopic murine Mitf

Further evidence for co‐regulation of a set of hypoxia‐regulated genes comes from examining the binding profiles of MITF under normoxia, versus the HIFs after 12 hr in hypoxia (Figure 4g). For most genes shown, binding by HIF1β is more robust than the other hypoxia‐induced factors, most likely in part a consequence of the antibody more efficiently pulling down HIF1β in the ChIP‐seq. We also detected some binding by HIF2α and HIF1β to many of the pigmentation genes (Figure 4h), raising the possibility that, in addition to MITF, these HIFs may directly contribute to any transient increase in expression of these genes under hypoxia (Figure 3b–d). However, we noted that the peak score of the HIFs on the pigmentation genes (Figure 4h) was considerably lower than known HIF targets such as BHLHE40 or SLC8A2 (Figure 4g) and consequently defining whether the HIFs contribute directly to the regulation of these genes will require further experimentation.

### 3.6 MITF represses its own expression

**Figure 5:**
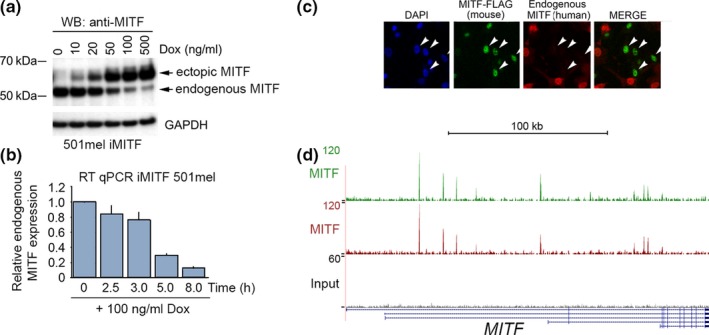
MITF represses its own expression. (a) Western blot showing doxycycline‐mediated induction of ectopic HA‐tagged MITF expression and corresponding expression of endogenous MITF. (b) qRT–PCR of the endogenous human MITF mRNA in 501mel cells induced to express ectopic murine HA‐tagged Mitf using 100 ng doxycycline. Expression normalised to ACTIN mRNA. (c) Immunofluorescence of 501mel cells transfected with an FLAG‐tagged mouse Mitf expression vector. Anti‐FLAG antibody was used to detect ectopically expressed Mitf WT and anti‐human‐specific mouse monoclonal antibody D5 used to detect endogenous MITF. (d) UCSC genome browser screenshot showing a biological replicate ChIP‐seq of HA‐MITF bound to the *MITF* locus

In the course of these experiments, we noted that induction of ectopic expression of HA‐tagged MITF led to repression of endogenous MITF mRNA (Table 1). Western blotting using anti‐MITF antibody to detect epitope‐tagged MITF induced by doxycycline together with endogenous MITF revealed that ectopic MITF expression potently repressed endogenous MITF levels (Figure 5a). This was confirmed by qRT–PCR of endogenous human MITF from cells in which ectopic mouse MITF was induced using doxycycline (Figure 5b). This result was further confirmed by using species‐specific antibody to detect endogenous human MITF by immunofluorescence in cells ectopically expressing mouse MITF (Figure 5c). As MITF could bind its own gene, as detected using ChIP‐seq (Figure 5d), the repression of MITF by its gene product was likely direct. Collectively, these data suggest that MITF participates in a negative feedback loop to suppress its own expression. These observations are important since a negative feedback loop invoking HIF1α was recently invoked to explain why MITF exhibits a dampened oscillation in skin melanocytes in response to UV irradiation (Malcov‐Brog et al., 2018). Our results suggest the feedback loop observed may also involve MITF‐mediated repression of MITF.

### 3.7 MITF controls succinate dehydrogenase to suppress pseudo‐hypoxia

**Figure 6:**
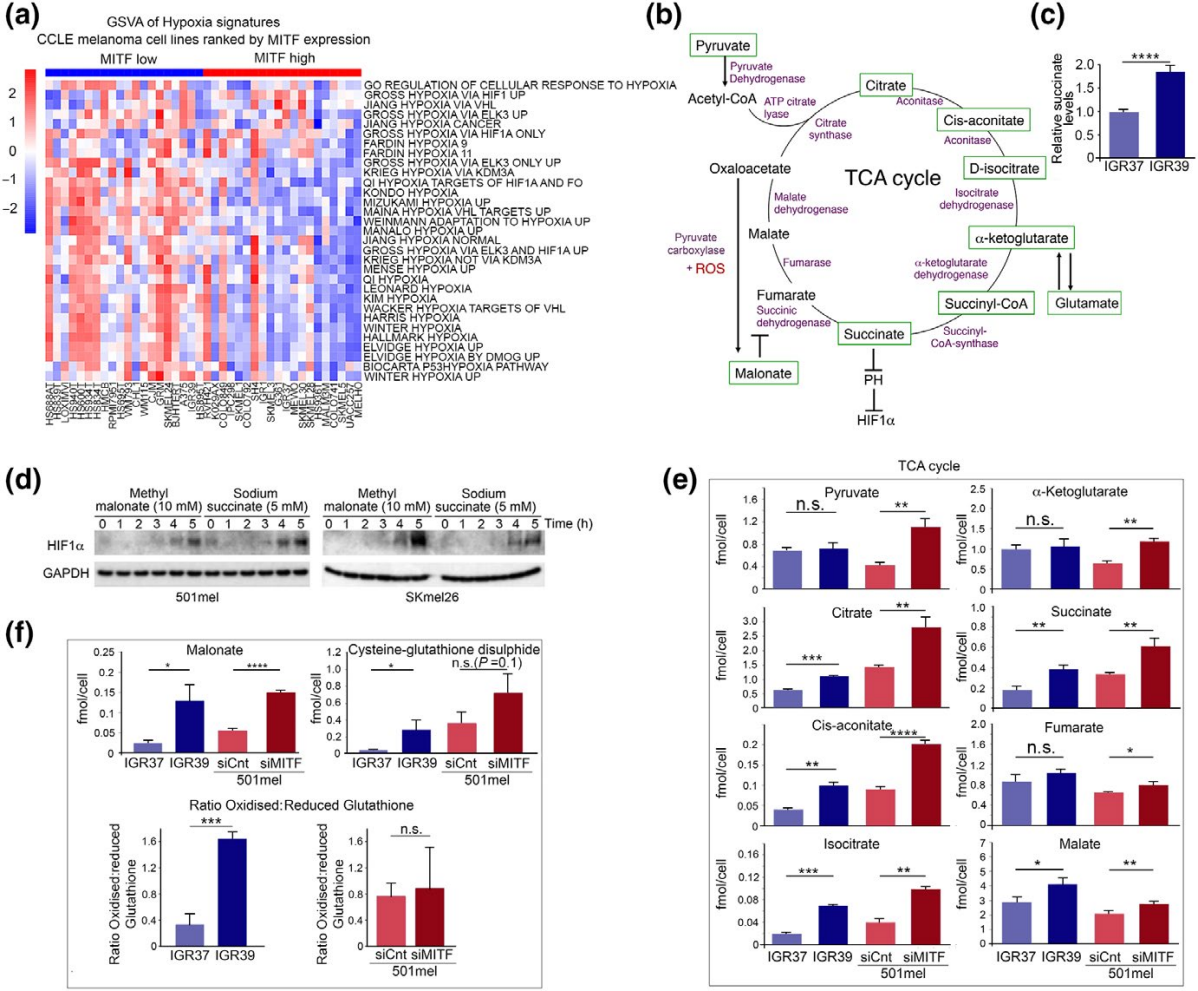
MITF regulates SDHB to suppress pseudo‐hypoxia. (a) GSVA analysis showing relative expression of indicated gene sets in the top and bottom 20 CCLE melanoma cell lines ranked by *MITF* expression. (b) The TCA cycle. Green‐boxed metabolites are significantly (*p* < 0.01) up‐regulated both in IGR39 compared to IGR37 cells and in MITF‐depleted 501mel cells compared to control. Succinate inhibits the prolyl hydroxylase (PH) that triggers HIF1α degradation. Malonate is generated from oxaloacetate by pyruvate carboxylase by the action of ROS. (c) Enzymatic determination of relative succinate levels in IGR37 versus IGR39 cells. *n* = 6 biological replicates of 2 technical replicates. ****p = < 0.0001. (d) Western blot showing expression of HIF1α in indicated cell lines treated over time with malonate or succinate as indicated. (e, f) Levels of indicated metabolites in IGR39 or IGR37 cells, or 501mel cells treated with control or MITF‐specific siRNA as indicated. For each metabolite data from each of three biological replicates is presented. Error bars indicate standard deviation, *n* = 3, *p = <0.05, **p = < 0.01, ***p = < 0.001, **** *p* = < 0.0001, n.s. = not significant, *t* test

**Figure 7:**
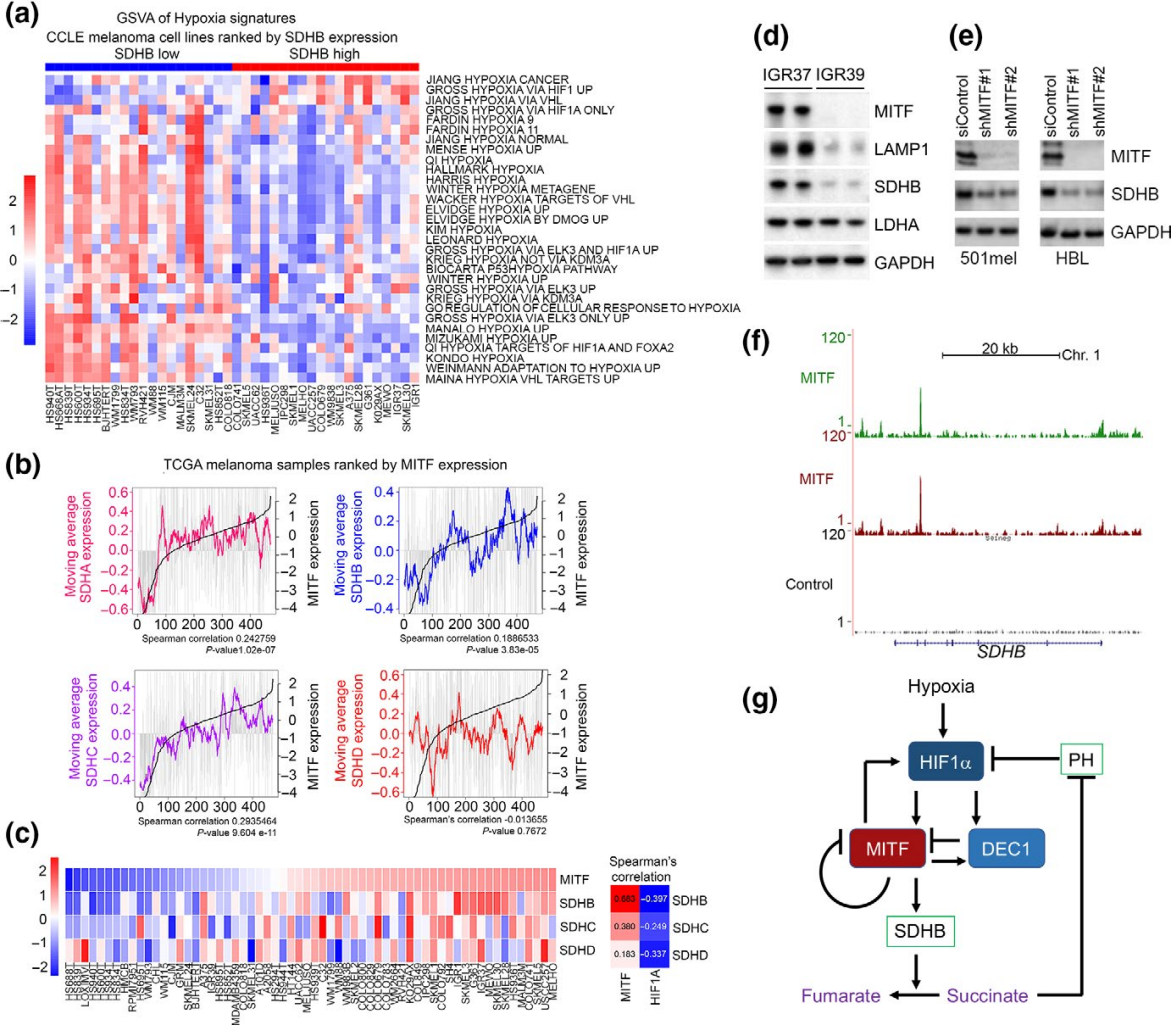
MITF regulates SDHB to suppress pseudo‐hypoxia. (a) GSVA analysis showing relative expression of indicated gene sets in the top or bottom 20 CCLE melanoma cell lines ranked by *SDHB* expression. (b) Analysis of TCGA human melanoma samples ranked by the MITF expression (black line) for expression of indicated SDH subunits. Gray lines indicate expression of SDH subunits in each melanoma sample. Colored lines indicate moving average of SDH subunit expression across each 20 melanoma samples. (c) Heatmap showing CCLE melanoma cell lines ranked by MITF expression and relative expression of indicated SDH subunits. Also shown is the Spearman's correlation between MIITF or HIF1α expression and each SDH subunit. (d) Duplicate Western blot of IGR37 and IGR39 cells using indicated antibodies. (e) Western blot of indicated cell lines transfected with control or MITF‐specific siRNA. (f) UCSC browser screenshot of a duplicate ChIP‐seq experiment showing MITF binding within the *SDHB* gene. (g) Diagram showing the interactions between the transcription factors HIF1α, DEC1 and MITF and the regulation of HIF1α by succinate. See text for details

The results so far suggest a complex relationship between MITF and the hypoxic response, with MITF able to affect the regulation of a set of hypoxia‐responsive genes in melanoma. Moreover, although low levels of MITF are also associated with a higher hypoxia gene expression signature in the TCGA melanoma cohort (Figure S1c), consistent with previous work (Cheli, Giuliano, et al., 2011a; Feige et al., 2011) indicating suppression of MITF in vivo in response to low oxygen, some cell lines, such as IGR39, appear to exhibit a constitutive hypoxia signature (Figure 1e, Figure S1j), even though grown under normoxic conditions. This suggests two distinct mechanisms operate to generate a hypoxia gene expression signature in melanoma: one dictated by low oxygen in the microenvironment, and the other by a cell intrinsic state. Significantly, GSVA analysis of the CCLE melanoma cell lines (Figure 6a) revealed that although there are some exceptions, in general a high constitutive hypoxia gene expression signature is associated with low levels of MITF mRNA. Since these cells are grown in normoxic conditions, the anti‐correlation between MITF and the hypoxia response cannot arise as a result of low oxygen. The origin of a constitutive or so‐called pseudo‐hypoxic gene expression profile has been attributed to elevated succinate levels (Selak et al., 2005). Succinate, a TCA cycle intermediate (Figure 6b) (Tretter, Patocs, & Chinopoulos, 2016), can inhibit the prolyl hydroxylase that triggers HIF1α protein degradation. Indeed, an enzymatic assay for succinate revealed that IGR39 cells contain approximately twofold higher levels than IGR37 cells (Figure 6c), consistent with succinate causing the IGR39‐associated pseudo‐hypoxia signature. Elevated succinate can arise either via reduced expression of succinate dehydrogenase (SDH) (Selak et al., 2005), a multi‐subunit complex that catalyzes the conversion of succinate to fumarate, or by inhibition of SDH by malonate that is generated by carboxylation of oxaloacetate by pyruvate carboxylase in cells undergoing oxidative stress (Reed, Ludwig, Bunce, Khanim, & Gunther, 2016). To ask whether a deregulated TCA cycle could be responsible for the pseudo‐hypoxia signature observed in some MITF‐low melanoma cell lines, we first confirmed that both malonate and succinate can lead to elevated HIF protein levels in melanoma cells (Figure 6d). We then used a mass spectrometry approach to interrogate the metabolite profile of the MITF‐high, non‐invasive melanoma cell line IGR37, and the IGR39 cell line derived from the same patient that is MITF‐low, invasive, and exhibits a pseudo‐hypoxic signature (Figure 1e). Since the increased pseudo‐hypoxia signature is predominantly associated with MITF‐low cell lines, we also profiled the MITF‐high cell line 501mel in which MITF was depleted using a specific siRNA. In focusing on TCA cycle metabolic intermediates, we found that several were elevated in the MITF‐low pseudo‐hypoxic IGR39 melanoma cells as well as in MITF‐depleted 501mel cells (Figure 6e; boxed metabolites in Figure 6b). These included succinate itself as well as the SDH inhibitor malonate (Figure 6f). Collectively, the metabolic profiling suggested a block to SDH activity arising either from inhibition of the enzyme or by reduced expression, leading to accumulation of several metabolic intermediates prior to fumarate in the TCA cycle. We also noted that IGR39 cells exhibited increased expression of cysteine–glutathione disulfide and a substantial increase in the ratio of oxidized: reduced glutathione (Figure 6f), consistent with elevated malonate being generated as a consequence of impaired control of oxidative stress in these cells. However, MITF depletion, that can also increase malonate levels, did not affect the oxidized: reduced glutathione ratio, indicating that MITF may affect succinate levels via an alternative pathway. Since MITF controls transcription, we first examined whether the mRNA expression of the key SDH subunit SDHB correlated with the pseudo‐hypoxic signature in the CCLE melanoma cell lines. The results suggested that pseudo‐hypoxia was linked to low SDHB mRNA expression in vivo (Figure 7a). Examining the TCGA melanoma cohort ranked by MITF expression (Figure 7b) also showed that those melanomas expressing especially low levels of MITF also exhibited reduced expression of the SDH subunits SDHA, B, and C, though no correlation was observed for SDHD. The correlation between SDHB and MITF was conserved in the CCLE cell lines (Figure 7c) where we did not detect significant mRNA expression of SDHA. We also noted that SDHB expression was low in the MITF‐low IGR39 cell line (Figure 7d) that exhibits elevated succinate levels compared to IGR37 cells (Figure 6c) and a pseudo‐hypoxia signature (Figure 1e). Significantly, MITF depletion using two different MITF‐specific siRNAs led to reduced SDHB expression in two melanoma cell lines (Figure 7e). Finally, ChIP‐seq revealed that MITF bound directly the *SDHB* gene (Figure 7f), but not to those encoding other SDH subunits (not shown). Collectively these results are consistent with MITF playing a key role in TCA cycle dynamics by activating SDHB expression to prevent accumulation of succinate and the consequent stabilization of HIF and generation of a pseudo‐hypoxia signature.

## 4 DISCUSSION

In vivo, melanoma cells transition though distinct phenotypic states in response to a changing microenvironment, and most notably can switch between invasive and proliferative phenotypes characterized by low and high levels of MITF activity respectively (Hoek et al., 2008; Hoek & Goding, 2010). Since melanoma cell lines isolated from human tumors tend also to fall into either proliferative or invasive, slow‐growing phenotypes (Hoek et al., 2006), it seems likely that established lines reflect specific phenotypic states within tumors (Tsoi et al., 2018), including those detected using single‐cell RNA‐seq (Rambow et al., 2018), that are then fixed and maintained under nutrient‐rich culture conditions where the microenvironmental stresses encountered in vivo are absent. This would be consistent with the observation that a number of cell lines in the CCLE cohort, as well as IGR39 cells exhibit gene expression programs associated with hypoxia even under normoxic conditions. By profiling the genomewide response and HIF‐DNA binding under hypoxia in 3 melanoma cell lines with different phenotypes, we were surprised to find only 23 genes up‐regulated by hypoxia in common between the three cell lines examined after 12 hr exposure to hypoxia. At later times, we see more genes that are commonly regulated, but beyond 12 hr a proportion of these genes is likely controlled indirectly. This extends observations in other cell types, and in melanocytes, that suggested that cells of different origin exhibit only a low degree of overlap in their hypoxia response signatures (Benita et al., 2009; Chi et al., 2006; Denko et al., 2003; Loftus et al., 2017; Widmer et al., 2013); our data reveal that even cells from the same tissue of origin or patient will respond differently to low oxygen, with only a relatively small number of core genes regulated by hypoxia in common between 3 different melanoma cell lines. If the different cell lines do indeed reflect different phenotypic states established in vivo, this result suggests that individual cells within tumors may respond very differently to hypoxia depending on their microenvironment‐induced phenotype. This nuanced response may be important in allowing cells to integrate their reaction to hypoxia with the impact of other microenvironmental cues with the aim of maximizing survival of the population as a whole. However, superimposed on different hypoxia‐induced gene expression programs in different cell lines is a core hypoxia response gene set in which genes implicated in glycolysis are highly represented in addition to *VEGFA,* a classic HIF target implicated in stimulating angiogenesis. The relative importance of the cell line‐specific hypoxia‐responsive genes in determining biological outcome remains to be determined, but a metabolic shift in response to hypoxia toward enhanced glucose uptake and glycolysis appears to represent a core hypoxia program (Semenza, 2013).

One important and unexpected result presented here is that in contrast to previous work (Cheli, Giuliano, et al., 2011a; Feige et al., 2011), we find that MITF is up‐regulated by HIF1α in response to hypoxia. Previous work has established that MITF is a key regulator of melanoma phenotype. Low MITF levels are associated with invasive (Carreira et al., 2006), tumor‐initiating (Cheli, Guiliano, et al., 2011b), and drug‐ and immunotherapy‐resistant phenotypes (Dugo et al., 2015; Konieczkowski et al., 2014; Landsberg et al., 2012; Muller et al., 2014; Riesenberg et al., 2015; Tirosh et al., 2016). It was not surprising therefore that hypoxia, a known trigger for invasiveness, was identified as a repressor of MITF expression (Cheli, Giuliano, et al., 2011a; Feige et al., 2011). The mechanism reported is indirect, with activation of HIF1α leading to up‐regulation of the transcription factor bHLHE40/DEC1, one of the common 23 genes we identify as induced in all three melanoma cell lines examined after 12 hr in hypoxia, and its consequent binding and repression of the MITF promoter. In contrast to this simple scenario, our results reveal a previously unsuspected complexity in the interplay between the hypoxic response and MITF (Figure 7g). Surprisingly, at early times, hypoxia induces the expression of MITF and its downstream target genes, an effect blunted in the presence of shRNA that prevents accumulation of HIF1α. The activation of MITF is likely to be directly mediated by the HIFs since ChIP‐seq analysis revealed that they bind upstream from the melanocyte‐specific MITF‐M promoter. Moreover, in response to hypoxia, the HIF‐mediated activation of genes implicated in glycolysis elevates glucose import and processing (Semenza, 2013). As shown here, and recently reported by others (Ferguson et al., 2017), glucose is required to maintain MITF expression. Thus, while hypoxia may ultimately lead to suppression of MITF, melanoma de‐differentiation and invasion, at early times MITF expression and that of its targets appear to be increased by a combination of direct activation of MITF expression by the HIFs, and most likely in part by the effect of HIF transcription factors maintaining intracellular glucose levels. In this respect, the combination of low oxygen together with variations in levels of key nutrients may significantly affect the kinetics and amplitude of the hypoxia and stress responses. We also note that in both our study and that of Feige et al. (2011), different melanoma cell lines exhibit different kinetics of response to hypoxia. Moreover, while Feige et al use 0.5% oxygen, our study uses 1% oxygen to induce hypoxia. Thus, differential responses between cell lines combined with different levels of oxygen used may explain why our results indicate that HIF can promote at least a transient increase in MITF expression, while other studies highlight an indirect role for HIF in suppressing MITF expression (Cheli, Giuliano, et al., 2011a; Feige et al., 2011).

Why should hypoxia induce MITF? Recent evidence indicates that although MITF may suppress expression of a pro‐invasive gene expression program and high MITF appears incompatible with survival of invasive cells (Falletta et al., 2017), low MITF may be insufficient to trigger invasion. This is most likely because in melanoma invasion reflects in part a response to a low nutrient supply environment (Falletta et al., 2017) and that by promoting proliferation, for example by activating CDK2 (Du et al., 2004), MITF imposes a high nutrient demand state that is incompatible with nutrient limitation. For example, 24 hr following glutamine deprivation MITF is suppressed to facilitate a switch to invasion (Falletta et al., 2017). However, at 4 hr following nutrient deprivation, MITF is activated to amplify the response to low nutrient levels. Thus, there are strong parallels between the response to low glutamine and low oxygen. MITF can activate expression of HIF1α (Busca et al., 2005) and PGC1α (Haq et al., 2013; Vazquez et al., 2013). It seems likely therefore that MITF’s role at early times following exposure to low oxygen is to amplify the hypoxia response with the aim to buy time for cells to maintain their gene expression and metabolic program in anticipation that oxygen supply may be rapidly restored. Consistent with this, MITF, like HIF1α, can up‐regulate *VEGFA*, though MITF binds at different sites, but can also activate a cohort of direct hypoxia‐regulated HIF target genes by binding the same sites as the HIFs. Only if oxygen levels remain low or are further reduced would MITF be down‐regulated by bHLHE40/DEC1, which is also up‐regulated by our inducible MITF and is directly bound by MITF (Feige et al., 2011) to enable cells to establish an invasive program. We found no evidence of an invasive gene expression signature in melanoma cell lines at 12 hr post‐hypoxia, a time when a range of HIF‐bound genes implicated in metabolic reprogramming were already induced. If oxygen levels remain low for an extended period, it seems likely that cells will reverse the initial up‐regulation of MITF expression.

We also establish a new metabolic role for MITF in controlling the TCA cycle, directly binding and regulating the gene encoding the key SDH subunit SDHB, with reduced MITF levels correlating to elevated succinate, a known inhibitor of the prolyl hydroxylase that promotes degradation of HIF1α (Selak et al., 2005). The regulation of *SDHB* by MITF provides a mechanism to promote a prolonged hypoxia response; the repression of MITF by bHLHE40/DEC1 would lead to decreased SDHB levels and consequently increased succinate that is able to inhibit the degradation of HIF1α. Although we have focused here on the impact of succinate on the hypoxia response, succinate is a key metabolic intermediate that plays a role in many biological processes (Tretter et al., 2016). These include mitochondrial ROS production, that may contribute in part to the elevated SDH inhibitor malonate in MITF‐depleted cells, the succinylation of proteins (usually on lysines), and epigenetic events including inhibition of histone demethylases and the ten‐eleven translocation family of 5‐methylcytosine hydroxylases (Tretter et al., 2016). Significantly, succinate has been termed an oncometabolite, with *SDHB* mutations being pathogenic (Saxena et al., 2016). As such, the low levels of SDHB in MITF‐low cells may contribute to disease progression in melanoma.

Finally, we reveal that MITF can repress its own expression. This negative feedback loop is likely important in restricting the activity of MITF, and in melanoma cells, enabling cells to maintain proliferation. It is also likely to contribute to the oscillations in MITF expression observed in vivo following UV irradiation (Malcov‐Brog et al., 2018).

In summary, by examining the genomic landscape of the hypoxic response in melanoma, our results have revealed a number of unanticipated features of the hypoxic response including transient up‐regulation of MITF by hypoxia, the self‐repression of MITF to provide a negative feedback loop, the limited repertoire of core hypoxia response gene in different melanoma lines, the regulation of MITF by glucose, and the ability of MITF to suppress the hypoxic response by controlling succinate levels via regulation of SDHB.

## CONFLICT OF INTEREST

The authors declare no conflict of interest.

## AUTHOR CONTRIBUTIONS

PL, IL, and CRG. conceived the project and designed and interpreted experiments. PL, IL, and PF performed experiments. TS undertook the metabolomic analysis. JC helped with bioinformatics analysis. DRM provided antibodies, FB and CRG provided resources and supervision, and PL, IL, and CRG wrote the manuscript.

5

## Data Availability

The RNA‐seq and ChIP‐seq datasets reported in this study have been deposited in the Gene Expression Omnibus (GEO) database, http://www.ncbi.nlm.nih.gov/geo (accession numbers GSE95280 and GSE132624).
