# Supplementary figures and images for: MITF controls the TCA cycle to modulate the melanoma hypoxia response
Source: Pigment Cell Melanoma Res. 2019 Jul 8;32(6):792–808. doi: 10.1111/pcmr.12802 (PMC6777998; doi:10.1111/pcmr.12802)

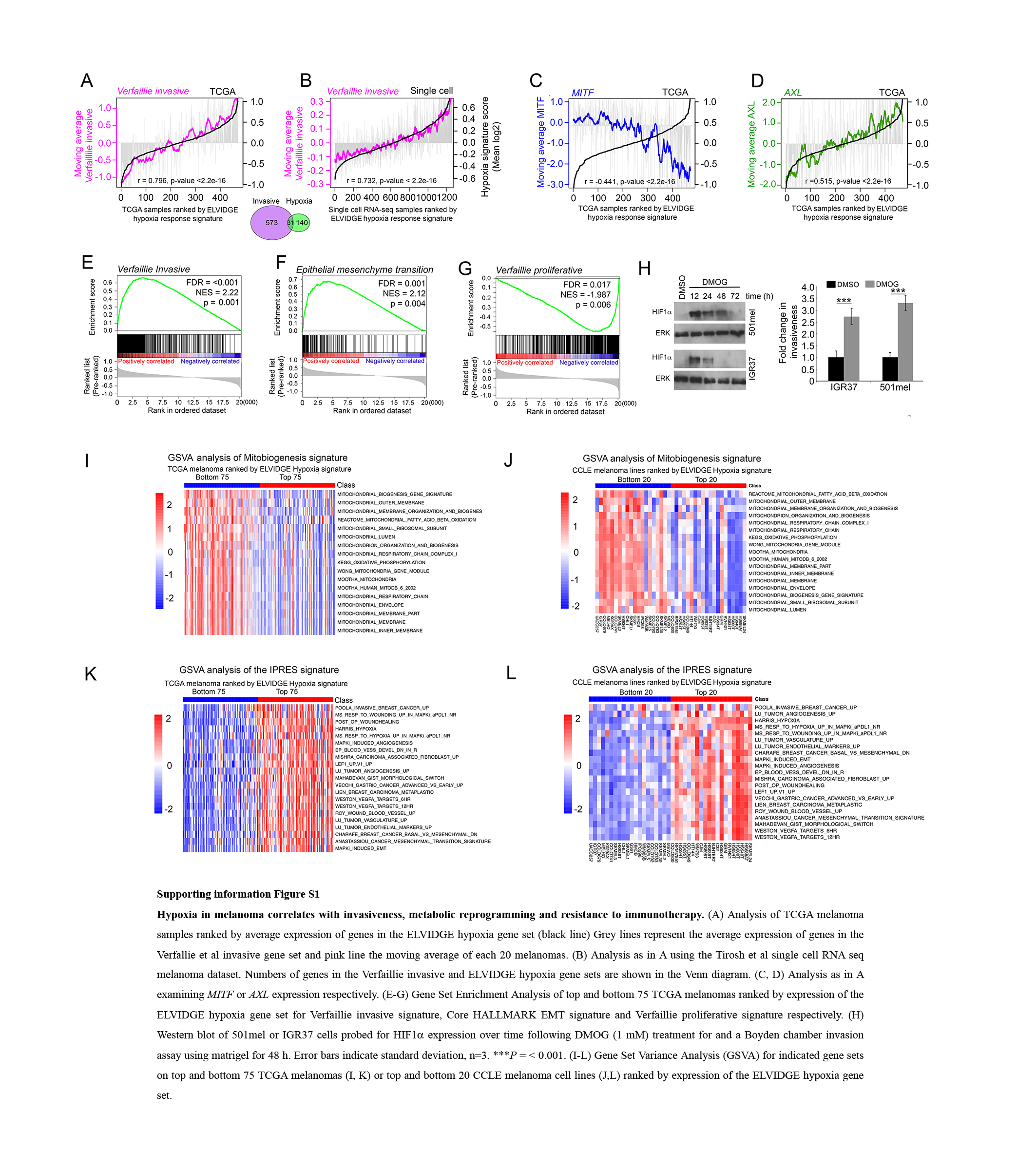

Supplement: Supplementary file 1 [file PCMR-32-792-s001.tif]

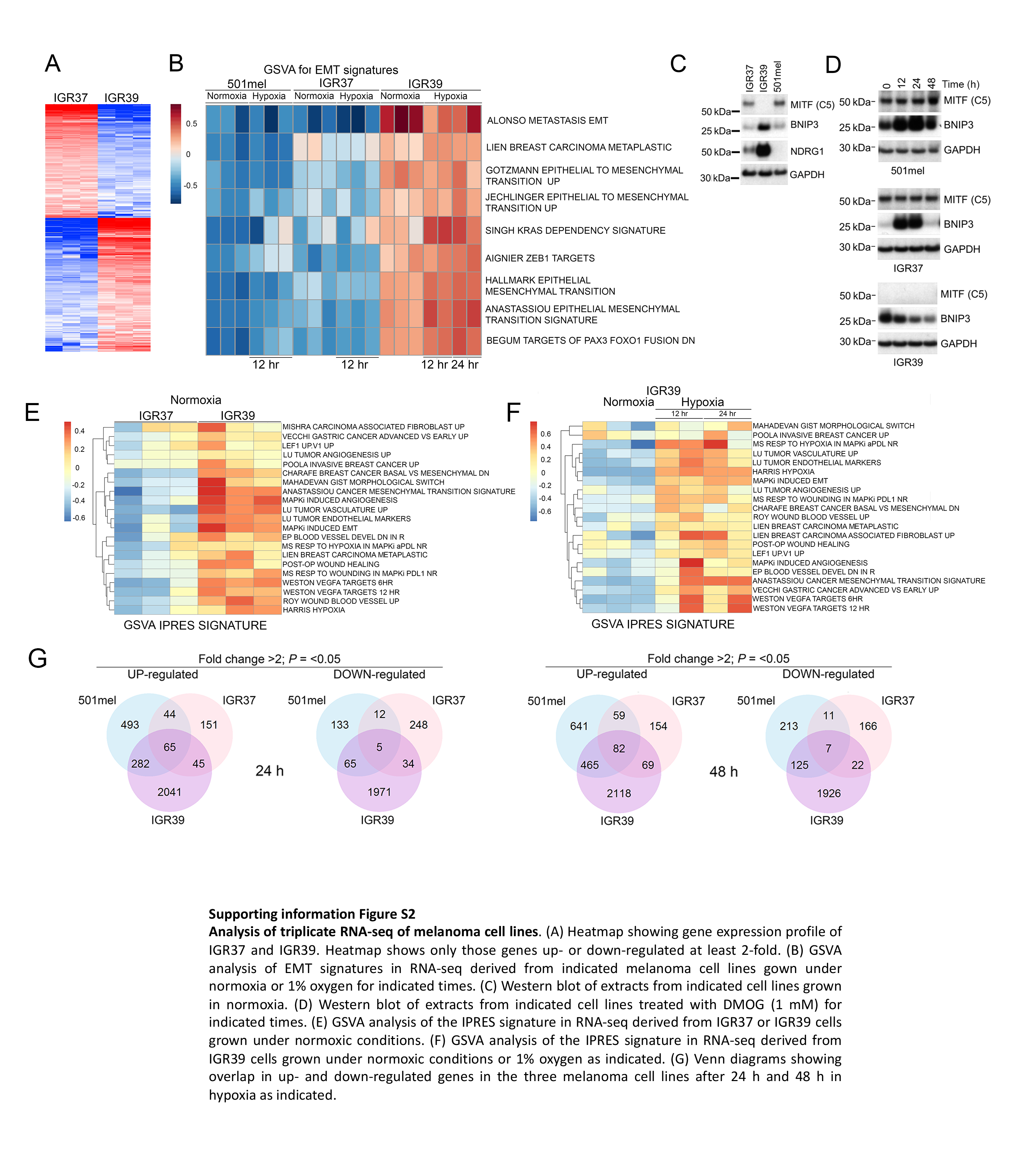

Supplement: Supplementary file 2 [file PCMR-32-792-s002.tif]

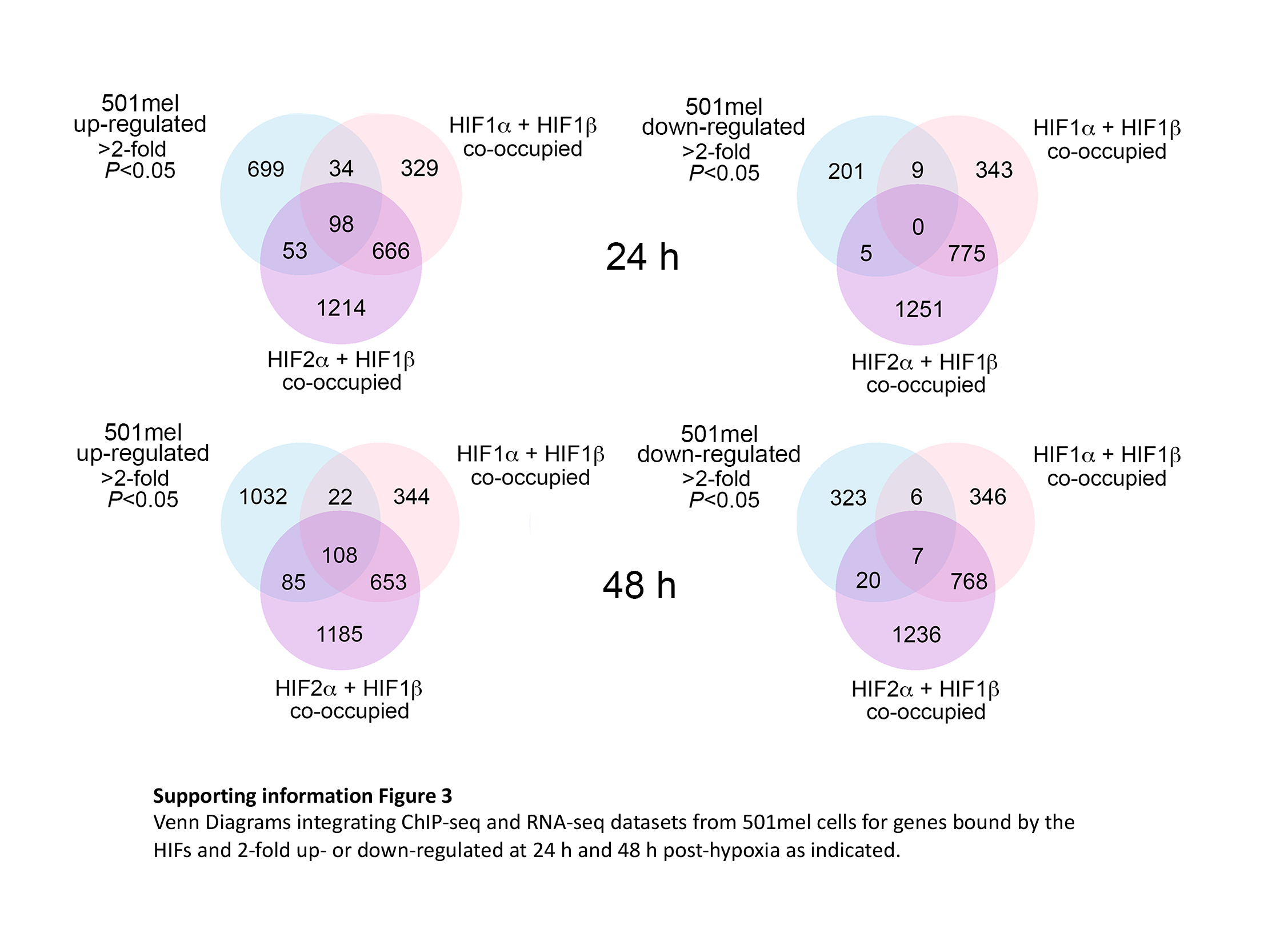

Supplement: Supplementary file 3 [file PCMR-32-792-s003.tif]

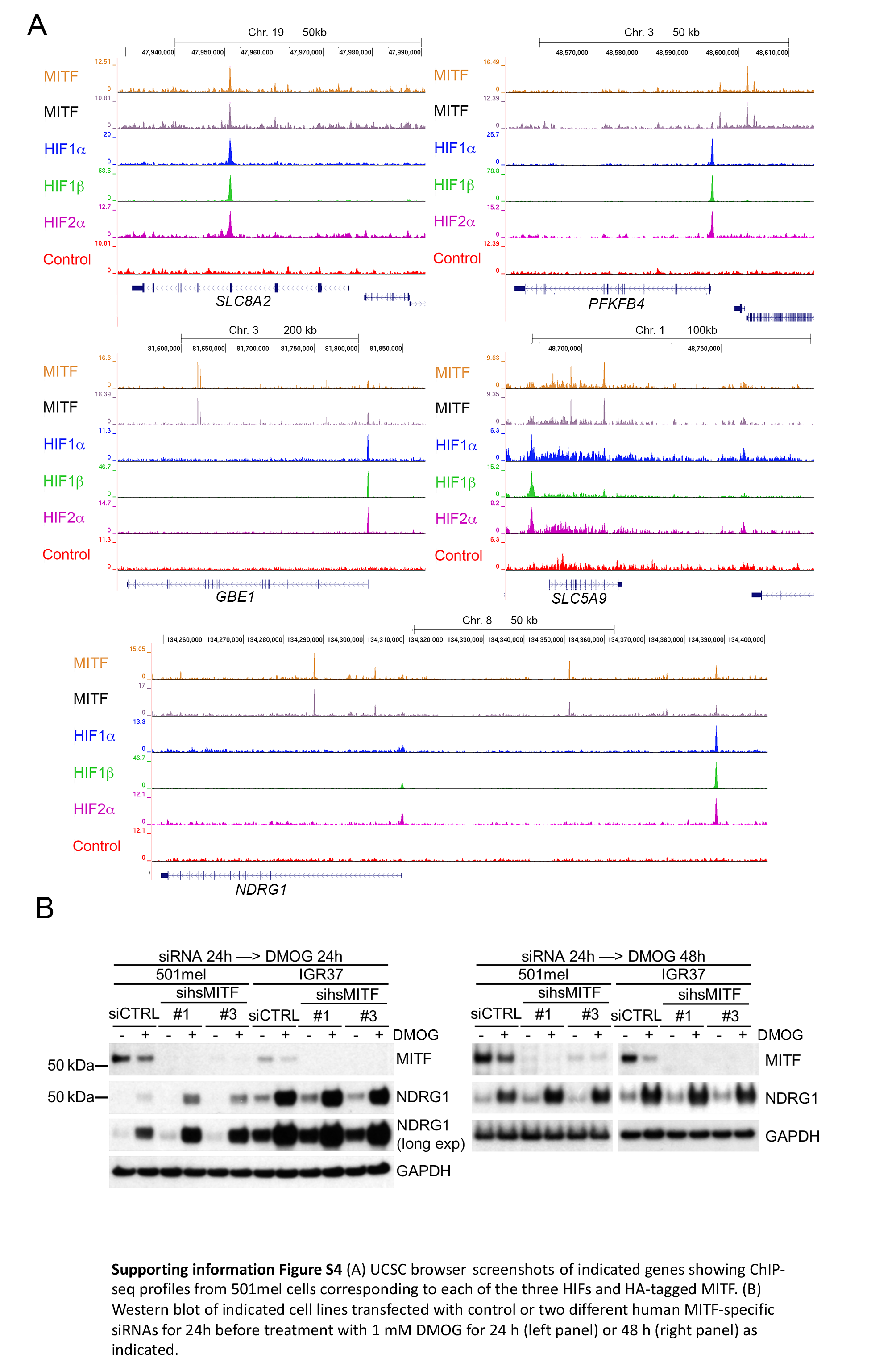

Supplement: Supplementary file 4 [file PCMR-32-792-s004.tif]
